# Loss of CHGA Protein as a Potential Biomarker for Colon Cancer Diagnosis: A Study on Biomarker Discovery by Machine Learning and Confirmation by Immunohistochemistry in Colorectal Cancer Tissue Microarrays

**DOI:** 10.3390/cancers14112664

**Published:** 2022-05-27

**Authors:** Xueli Zhang, Hong Zhang, Chuanwen Fan, Camilla Hildesjö, Bairong Shen, Xiao-Feng Sun

**Affiliations:** 1 Institute of Medical Sciences, School of Medicine, Örebro University, 702 81 Örebro, Sweden; zhangxueli@gdph.org.cn (X.Z.); hong.zhang@oru.se (H.Z.); 2 Department of Ophthalmology, Guangdong Eye Institute, Guangdong Provincial People’s Hospital, Guangdong Academy of Medical Sciences, Guangzhou 510080, China; 3 Department of Oncology and Department of Biomedical and Clinical Sciences, Linköping University, 581 83 Linköping, Sweden; xuntian2005@163.com (C.F.); Camilla.Hildesjo@liu.se (C.H.); 4 Institute for Systems Genetics, Western China Hospital, Sichuan University, Chengdu 610017, China; bairong.shen@scu.edu.cn

**Keywords:** CHGA, protein biomarker, colon cancer, diagnosis, machine learning, tissue microarrays

## Abstract

**Simple Summary:**

The identification of effective novel biomarkers is emergently needed in colon cancer patients. In the present study, firstly we predicted that CHGA could be a biomarker for colon cancer based on the protein–protein interaction network of all the reported biomarkers that were collected from our colorectal cancer biomarker database (CBD). Then we verified our results using a diagnostic test in gene expression data and an immunohistochemistry test. The results of this study suggest that a loss of CHGA expression from the normal colon and adjacent mucosa to colon cancer may be used as a valuable biomarker for early diagnosis of colon cancer patients.

**Abstract:**

Background. The incidence of colorectal cancers has been constantly increasing. Although the mortality has slightly decreased, it is far from satisfaction. Precise early diagnosis for colorectal cancer has been a great challenge in order to improve patient survival. Patients and Methods. We started with searching for protein biomarkers based on our colorectal cancer biomarker database (CBD), finding differential expressed genes (GEGs) and non-DEGs from RNA sequencing (RNA-seq) data, and further predicted new biomarkers of protein–protein interaction (PPI) networks by machine learning (ML) methods. The best-selected biomarker was further verified by a receiver operating characteristic (ROC) test from microarray and RNA-seq data, biological network, and functional analysis, and immunohistochemistry in the tissue arrays from 198 specimens. Results. There were twelve proteins (MYO5A, CHGA, MAPK13, VDAC1, CCNA2, YWHAZ, CDK5, GNB3, CAMK2G, MAPK10, SDC2, and ADCY5) which were predicted by ML as colon cancer candidate diagnosis biomarkers. These predicted biomarkers showed close relationships with reported biomarkers of the PPI network and shared some pathways. An ROC test showed the CHGA protein with the best diagnostic accuracy (AUC = 0.9 in microarray data and 0.995 in RNA-seq data) among these candidate protein biomarkers. Furthermore, immunohistochemistry examination on our colon cancer tissue microarray samples further confirmed our bioinformatical prediction, indicating that CHGA may be used as a potential biomarker for early diagnosis of colon cancer patients. Conclusions. CHGA could be a potential candidate biomarker for diagnosing earlier colon cancer in the patients.

## 1. Introduction

Colon cancer contributes to cancer mortality and morbidity [1]. In 2020, there were 104,610 new colon cancer cases and 53,200 deaths that are caused by colon cancer in the United States, estimated by the National Cancer Institute [2]. Surgery is the primary treatment for early-stage colon cancer [3]. With the development of modern medicine and surgery technology, the five-year survival rate of stage I and II has increased to more than 90% [4]. However, the rate of stage IV is around 10% [4]. What’s more, more than 50% of patients are already at late-stage colon cancer when they are diagnosed [5]. As such, the timely and accurate early diagnosis of colon cancer is highly needed.

Biomarkers are biological indicators for special clinical conditions or states, which have been reported many times, improving the diagnosis of colon cancer [6,7]. In previous work, our research group has established an integrated colorectal biomarker database (CBD), which has collected all the colon cancer-related biomarkers [7]. However, few of these biomarkers have been used in clinical practise, and the effects are not convincing [7,8]. Hence, it is needed to predict new biomarkers. Recently, more and more studies have suggested that combining different single biomarkers together as multiple biomarkers could reach better clinical performance than single biomarkers [9,10,11]. Therefore, the development of multiple biomarkers could be a new direction in biomarker discovery.

Colon cancer and rectal cancer have many similar features in both genotype and phenotype, which are always grouped as colorectal cancer (CRC) [12]. It has been suggested that colon cancer and rectal cancer share many biomarkers [7]. Hence, the application of new colon cancer biomarkers in rectal cancer can be expected. The development of cancer is a continuous process. Many studies report that some diagnosis biomarkers can also serve as prognosis biomarkers in CRC [13,14], which are considered as multiple-functional biomarkers. As such, the expansion of novel diagnosis biomarkers in prognosis is reasonable.

Network topology analysis is an important component of system biology study [15]. Many researchers have proven that biomarkers occupy specific positions on biological interaction networks [16,17,18]. Based on this theory, we predicted three novel miRNA biomarkers for colorectal cancer diagnosis, using network topology features from the miRNA-mRNA interaction network, and they showed good diagnosis value in the verification test by meta-analysis [18]. Proteins are a major part of colon cancer biomarkers [7]. The String database contains highly credible human protein–protein interaction (PPI) networks that were collected from different resources, which could be the effective data source for protein-related network topology analysis [19].

Machine learning (ML) has been applied in bioinformatics and complex network analysis for many years [20]. Support vector machine (SVM) is a supervised-based ML method focusing on classification and regression analysis, which has been developed as a popular method in bioinformatics since it has good accuracy and robustness [21]. Several published studies utilized SVM and PPI networks in cancer biomarker prediction [22,23,24]. However, none of them used identified biomarkers for the training dataset [22,23,24], which we think decreased the prediction credibility.

With its high robustness, low heterogeneity, and extensive adaptability, bioinformatics (dry lab) experiments have become a new focus in the biomedicine field, especially in cancer biomarker discovery [25]. Traditional biomedicine (wet lab) experiments are closer to the actual situation, which is suggested with high credibility. In the past years, our research group predicted and identified several useful CRC biomarkers using traditional biomedicine experiments or bioinformatics [17,18,26,27].

Chromogranin A or parathyroid secretory protein 1 (gene name *CHGA*) is a member of the grain family of neuroendocrine secretory proteins, and it is located in secretory vesicles of neurons and endocrine cells such as islet beta-cell secretory granules in the pancreas [28]. In humans, chromogranin A protein is encoded by the *CHGA* gene.

In the present study, we used the reported colon cancer diagnostic biomarkers to predict new biomarkers via ML methods, based on the topology features from PPI network. Diagnostic receiver operating characteristic (ROC) test, immunohistochemistry (IHC), and biological network and function analysis were conducted to make the verification and confirmed that CHGA could be a future biomarker for colon cancer diagnosis. Meanwhile, the multiple biomarkers consisting of the 12 predicted biomarkers have been suggested with high diagnostic accuracy. Further, the diagnosis and prognosis value of CHGA in both colon and rectal cancer were evaluated, which indicated that CHGA could be a promising diagnostic biomarker but not a prognostic biomarker in CRC.

## 2. Materials and Methods

### 2.1. Patients’ Information

The present study included 198 specimens consisting of 22 biopsy primary tumors, 55 surgical primary tumors, 22 metastatic lymph nodes, 46 adjacent normal mucosa specimens (adjacent to the primary tumor on the same histologic section), and 53 distant normal mucosa specimens from the proximal or distal margin (4–35 cm from the primary tumor) of the resected colorectum. All the patients were from the Southeast Swedish Health Care region, Sweden. The detailed characteristics of the patients and specimens are presented in Table 1. All the specimens were paraffin-embedded and fabricated into tissue microarray (TMA) as the previous description [29]. The study was conducted in accordance with the Declaration of Helsinki, and the protocol was approved by the institutional review board of Linköping University, Sweden (Dnr-2012-107-31, Dnr-2014-79-31).

### 2.2. The Measurements of CHGA Expression by IHC

CHGA expression was determined by IHC on 5-μm TMA sections as described previously [29]. Briefly, the sections were deparaffinized, rehydrated, and masked epitope retrieval. Then, after blocking the activity of endogenous peroxidase, the sections were incubated with the CHGA monoclonal rabbit anti-human IgG (CM10C, BIOCARE MEDICAL) in a 1:100 dilution with antibody dilution buffer overnight. The next day, the sections were washed in PBS and then incubated with Envision System Labelled Polymer-HRP anti-rabbit (Dakocytomation (Glostrup, Denmark) for 30 min. Next, the sections were subjected to 3,3′-diaminobenzidine tetrahydrochloride for 8 min and then counterstained with haematoxylin. Negative and positive controls were included in each staining run. CHGA expression on all the slides were scored by two independent investigators: 0, no staining; +1, ≤2% staining in the normal intestinal or tumor cells; and +2, >2% staining in the normal intestinal or tumor cells [30].

### 2.3. Data Collection

We downloaded the colon cancer differential expression (DE) data from the GEPIA (Gene Expression Profiling Interactive Analysis) database (http://gepia.cancer-pku.cn/index.html accessed on 20 May 2020), which concluded normalized and comprehensive high-throughput RNA sequencing (RNA-Seq) data from the Cancer Genome Atlas (TCGA) and Genotype-Tissue Expression (GTEx) database. A total of 275 colon cancer patients and 349 normal controls were included. A linear model and the empirical Bayes method were used to calculate the DE genes (DEGs) by the limma package in R. *p*-value < 0.05, and |Log2FC| > 1 was selected as the cut off for the DEGs. All the DEGs, along with their statistics results, can be found in the Table S1.

The Human PPI network (confidence > 0.7) was downloaded from the String database via the NDEx public server. Colon cancer diagnostic protein biomarkers were downloaded from the CBD database (Table S2).

The Gene Expression Omnibus (GEO) database provided the microarray data named “GSE 44861” for verification of candidate biomarkers, which contained 111 colon tissues from tumors and adjacent noncancerous tissues. These GE data were from GPL3921 Platform.

### 2.4. Colon Cancer Specific PPI Network Construction

The colon cancer DEGs were transferred to protein by searching in the NCBI protein database then mapped to the Human PPI network. The greedy search algorithm of jActiveModules in Cytoscape was used to find the most highly scored subnetwork from the human PPI network according to colon cancer patients DE genes’ *p*-value. In the greedy searching, firstly every *p*-value of DE gene was transferred to a *z*-score using the Stouffer’s *Z*-score model based on the inverse normal cumulative distribution. A smaller *p*-value will have a higher *z*-score, which means that the genes with higher *z*-scores are more related to colon cancer. Then a *k*-subnetwork will be given a *z*(A):

$$z\left(A\right)=\frac{1}{\sqrt{k}}\underset{i\in A}{\sum }z_{i}$$

where *z_i_* is a random gene, and k is the number of genes, on the subnetwork. We selected the subnetwork with the highest summary *z (A)* after ten iterations. This subnetwork was constructed by every highly scored DE genes along with one of its neighbor genes. Here we named this subnetwork as colon cancer-specific PPI network (CCS-PPIN).

Several network topology features were selected from the CCS-PPIN: Average shortest path length, betweenness centrality, closeness centrality, clustering coefficient, degree, eccentricity, neighborhood connectivity, number of directed edges, radiality, stress, and topological coefficient. The definition of these network features was shown in Table S3. Table S4 offers the model topology features for the CCS-PPIN.

### 2.5. Prediction Model Construction

A total of 31 diagnostic protein biomarkers that were collected from the CBD database were found on the CCS-PPIN. A total of 31 non-DE proteins in the CCS-PPIN were randomly selected as the control group. We took 22 biomarkers and 22 non-DE proteins as the training set to establish an SVM model to predict biomarkers and another nine biomarkers and nine non-DE proteins as the test set to test the model performance. Supplementary Materials Table S5 presents the dataset for machine learning model construction.

A regression tree is an ML method that combines the advantages of a decision tree and regression. The aim of the regression tree is to find the best features and their cut off to classify the target. The regression tree that is implemented by R package “rpart” was utilized to choose the useful network features to distinguish the biomarkers from non-biomarkers. We inputted the 11 original calculated network features together with the category information of biomarkers and non-biomarkers in the training datasets, into the regression tree model. Then the model selected the best network features, which could best distinguish the biomarkers and non-biomarkers. The selected features would be used as the input features for the SVM prediction model.

SVM is a popular supervised machine learning method for classification issues. SVMs can efficiently perform nonlinear classification using the so-called kernel trick, which implicitly maps its inputs into a high-dimensional feature space. Using the selected features by regression tree, SVM was used to construct the topology model to predict new biomarkers in the CCS-PPIN, which was conducted by R package “kernlab”. The biomarkers and non-biomarkers in the training data were used to construct the model and test data was used to evaluate the model performance. We tried eight different kernels to train the SVM model to get the best prediction accuracy. The area under the curve (AUC) on the receiver operating characteristic (ROC) curve was used as an indicator to evaluate the models. A total of four kernels showed good performance (AUC > 0.7): Bessel with an AUC of 0.765; Spline with an AUC of 0.728; Hyperbolic tangent and ANOVA RBF with an AUC of 0.716. Another two kernels showed normal performance (AUC > 0.6): Radial Basis and Laplacian. Polynomial and Linear showed low performance. (AUC < 0.6) Finally, Bessel kernel was chosen as the Kernel function in the SVM. A total of 2401 DE proteins in the CCS-PPIN was selected to predict new biomarkers. (Table S6).

### 2.6. ROC Test for the Predicted Biomarkers

The ROC curve was used to identify the predicted biomarkers from the SVM model using the patients’ data that was provided by the GSE 44,861 microarray data. The AUC of the ROC curve was recorded to compare the diagnostic accuracy of candidate biomarkers.

### 2.7. PPI Network and Biological Function Analysis

PPI network analysis, Gene Ontology (GO) annotation, and KEGG pathway enrichment that were performed by String and Gluego on Cystoscope were conducted to analyze the candidate biomarkers that were calculated from our SVM model and confirmed biomarkers from the CBD database in biological interaction and function level.

### 2.8. Multiple Biomarkers Identification

The predicted biomarkers were collected to combine as multiple biomarkers by logistic regression. The ROC curve was drawn to test the diagnostic accuracy of multiple biomarkers.

## 3. Results

**Figure 1:**
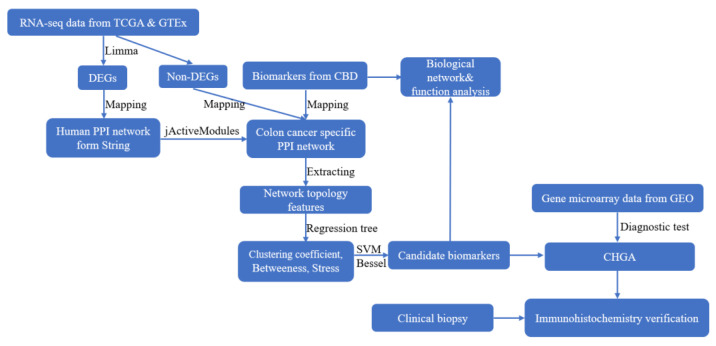
Schematic flow chart of the present study for early diagnosis of colon adenocarcinoma. The starting materials were derived from the RNA seq data in the TCGA and GTEx databases. Differential expressed analysis (DEA) between the colon cancer patients and normal controls was conducted. The differential expressed (DE) genes were then mapped to the Human PPI network (from String) to construct a colon cancer-specific PPI network, and machine learning was used to predict new potential biomarkers based on the network features of the confirmed biomarkers from our CBD database. The diagnostic test (ROC test) of the predicted biomarkers were further verified in GEO microarray data. The candidate biomarker (CHGA) was finally confirmed by immunohistochemistry tissue microarrays.

Figure 1 shows the analysis pipeline for this study.

### 3.1. Colon Cancer Specific Protein-Protein Interaction Network (CCS-PPIN)

**Figure 2:**
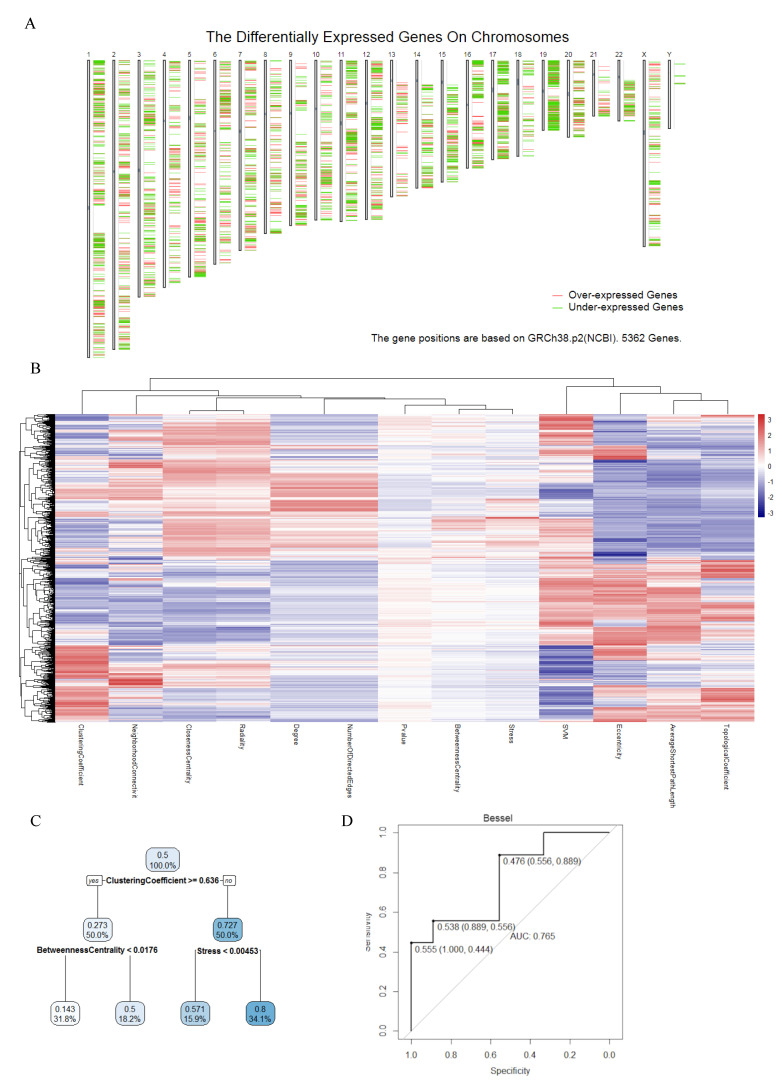
(**A**) The differential expressed (DE) genes on various chromosomes. (**B**) Performance of network features. (**C**) Regression tree in biomarker prediction model construction. Clustering coefficient, betweenness centrality, and stress were selected as the features for the next SVM model. (**D**) Bessel showed the best prediction accuracy in the ROC test (AUC = 0.765).

In total, 5562 colon cancer DEGs were identified based on the *p*-value and Log2FC. Figure 2A shows the DEGs position on chromosomes. After mapping these DEGs using jActiveModules, we got the colon cancer-specific protein–protein interaction network (CCS-PPIN). CCS-PPIN contains 9624 nodes and 199,553 edges. A total of 11 original network topology features (average shortest path length, betweenness centrality, closeness centrality, clustering coefficient, degree, eccentricity, neighborhood connectivity, number of directed edges, radiality, stress, topological coefficient) of each node were extracted from the CCS-PPIN, and their overall performance was shown on Figure 2B.

### 3.2. Machine Learning Based Biomarker Prediction

A regression tree was conducted to select useful parameters for the SVM model among the 11 original network features. Finally, the clustering coefficient, betweenness centrality, and stress were selected (Figure 2B).

**Figure 3:**
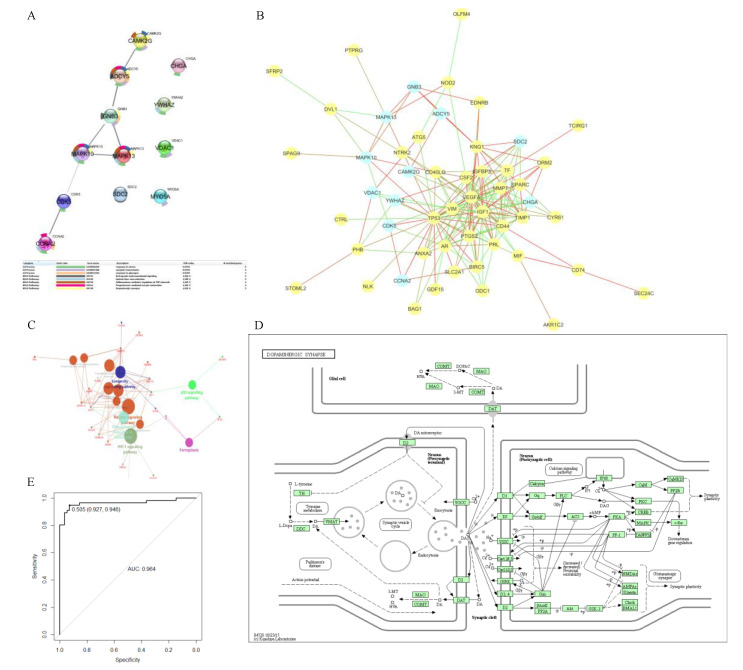
PPI network and biological function analysis of the predicted biomarkers. (**A**) PPI relationships and enriched pathways (table) for the 12 predicted biomarkers. A total of seven predicted biomarkers related to each other. In addition, nine predicted biomarkers were mapped on the response to stress pathway. The candidate biomarkers that had a strong relationship were mapped in the same pathway. (**B**) PPI network for predicted and confirmed biomarkers. The color of lines represented the confidence of connected evidence: the closer to red, the higher evidence. Generally, the predicted and confirmed biomarkers had strong relationships with each other; specifically, not as the other ten predicted, which are connected closely, CHGA and SDC2 were separated from them and were hubs in their belonged small networks. (**C**) KEGG pathway enrichment analysis results for predicted and confirmed biomarkers. Big circles represent enriched pathways, and small circles/diamonds represent confirmed/predicted biomarkers. Pathways and biomarkers were connected if the biomarkers were mapped on the pathways. There were some overlapping pathways among the confirmed and predicted biomarkers, and five of them were mapped on the Dopaminergic synapse pathway. (**D**) Dopaminergic synapse pathway. (**E**) Diagnostic ROC curve for multiple biomarkers combined by the 12 predicted biomarkers, which showed an AUC of 0.964.

We tried different kernels to train the SVM model using training data and predict the test data. ROC curve was selected to calculate the perdition accuracy (Figure 2C). With its 0.765 prediction AUC, Bessel was selected as the kernel for the final biomarker prediction SVM model. Figure 2D shows there were 2401 DE proteins on the CCS-PPI, which was selected to predict new colon cancer biomarkers using the SVM model. Through the model, each protein will be given a point, which is the possibility to be a biomarker. We set 0.99 as a cutoff for the SVM point, and Figure 3A presents the 12 predicted biomarkers.

### 3.3. Verification of Predicted Biomarkers

ROC analysis that was performed by gene expression data was used to test the diagnostic value of candidate biomarkers that were predicted by the SVM model. A total of 11 predicted biomarkers were found on the GPL, and they all showed high AUC (bigger than 0.5). Among them, CHGA had the best AUC (0.9). Table S6 lists all the tested proteins (DEGs) along with their SVM point and diagnostic AUC.

**Figure 4:**
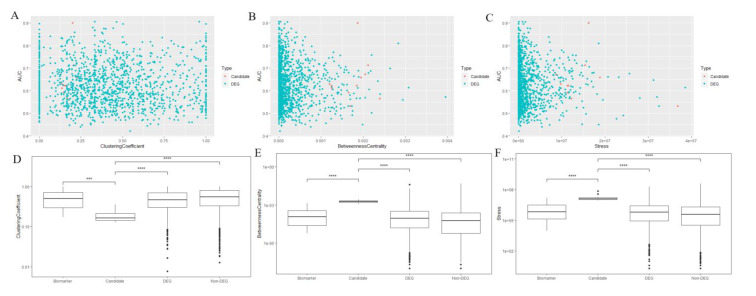
Comparison of the network features of predicted biomarker candidates with other genes. (**A**–**C**): Cluster coefficient (**A**), betweenness centrality (**B**), and stress (**C**) of candidate biomarkers (red points) and DEGs (green points) on CCS-PPIN. Biomarker candidates showed specific features in Cluster coefficient (0.125–0.25) and betweenness centrality (0.001–0.0025). (**D**–**F**): Boxplot of cluster coefficient (**D**), betweenness centrality (**E**), and stress (**F**) of the identified biomarkers, biomarker candidates, DEGs, and non-DEGs. Biomarker candidates showed significant differences with other genes.

Scatterplots and boxplots of the network features of the predicted model for the predicted biomarkers and other genes are shown in Figure 4. Significant differences were identified among the predicted/identified biomarkers and other genes.

### 3.4. PPI Network and Biological Function Analysis for Predicted Biomarkers

We used the String database to explore the relationship between these predicted biomarkers in the PPI network and biological pathways. Figure 3A shows the PPI network and biological function analysis results of 12 predicted biomarkers. CCNA2, CDK5, MAPK10, MAPK13, GNB3, ADCY5, and CAMK2G showed a strong relationship in the PPI network. KEGG pathway enrichment analysis showed that five of them were mapped on the Dopaminergic synapse pathway. (Figure 3D) According to the GO annotation, nine of these predicted biomarkers were related to the response to stress. 

### 3.5. Relationship for Reported and Predicted Biomarkers on PPI Network and Biological Function

In order to investigate the relationship between the already reported biomarkers from the CBD database and the newly predicated biomarkers, we mapped them together in the human PPI network (Figure 3B). We found that most of the predicted biomarkers were the close neighbors of confirmed ones, and some famous biomarkers such as TP53, VEGFA, and IGF1 were still hubs for this PPI. A total of 10 predicted biomarkers had direct relationships with each other but not SDC2 or CHGA, which occupied two separate positions beside others. What’s more, from Table 1, we found that SC2 and CHGA had the highest AUC (0.71 and 0.90) on the ROC curve of the diagnostic test.

We performed the KEGG pathway enrichment analysis for the confirmed and predicted biomarkers and mapped them together with the results in Figure 3C. There were two overlapping for the two group biomarkers: Inflammatory mediator regulation of TRP channels, progesterone-mediated oocyte maturation. The p53 signalling pathway and Ferroptosis were the two most confirmed biomarker mapped pathways, and GnRH signalling pathway was the most mapped pathway for only predicted biomarkers.

GO annotation in biological process, Cellular component, Immune system process and Molecular function level were conducted (Table S7), and we found three overlapping pathways for the confirmed and predicted biomarkers: Positive regulation of osteoblast differentiation, morphogenesis of an epithelial sheet, positive regulation of fibroblast proliferation, and regulation of fibroblast proliferation. CCNA2, as a predicted biomarker, was mapped on all these four pathways.

### 3.6. Identification of Multiple Biomarker

We combined the predicted biomarkers as multiple biomarkers via logistic regression and using AUC analysis to test its diagnostic value. The ROC on the AUC curve of multiple biomarkers was 0.964 (Figure 3E).

### 3.7. Verification for CHGA

**Figure 5:**
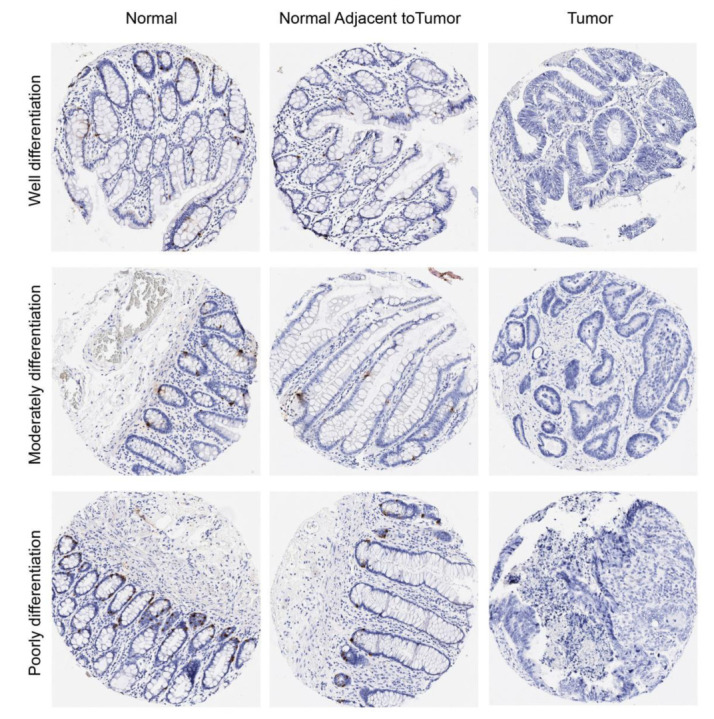
Protein expression of CHGA in normal mucosa, normal adjacent mucosa, and tumor from the same patient with colon cancer. The CHGA protein was positively expressed in the normal, and adjacent mucosa (the brown colour) and absolutely lost the CHGA expression in the tumor regardless of well-, moderately-, or poorly-differentiated cancers. Magnifications 10× and 40×.

With its best performance in a diagnostic test, CHGA was selected to make further verification. Figure 5 presents the IHC results for CHGA in the CRC TMA samples. The CHGA protein was positively expressed in the normal colon and adjacent colon mucosa (the brown colour) and lost the CHGA expression in the CRC regardless of well, moderate, or poor differentiation of the cancers.

**Figure 6:**
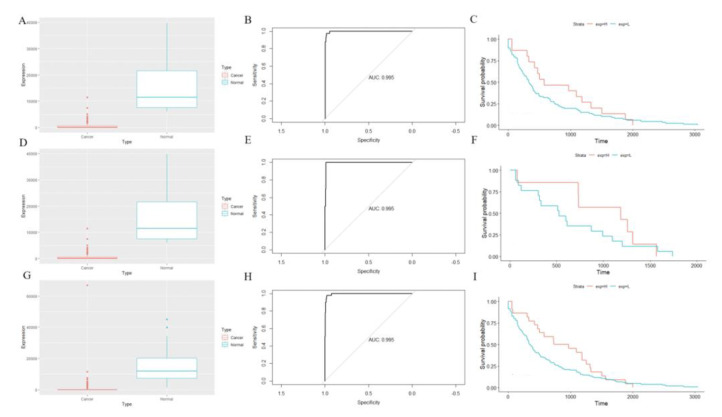
CHGA expression distribution of colon cancer (**A**), rectal cancer (**D**) and CRC (**G**) compared with normal controls. CHGA had a significant difference in cancers with normal controls. Diagnostic ROC test for CHGA in colon cancer (**B**), rectal cancer (**E**), and CRC (**H**). With AUCs of 0.995, GHGA showed a high potential of being a good diagnostic biomarker in CRC. Survival curves of CHGA in colon cancer patients (**C**), rectal cancer patients (**F**), and CRC patients (**I**). CHGA performed poorly in the prognosis of CRC (*p* value = 0.24, 0.38, and 0.13, respectively).

Figure 6 shows the CHGA expression in normal controls and cancer patients (Figure 6A: colon cancer patients, Figure 6D: rectal cancer patients, Figure 6G: CRC patients), diagnostic ROC tests (Figure 6B: colon cancer patients, Figure 6E: rectal cancer patients, Figure 6H: CRC patients), and survival tests (Figure 6C: colon cancer patients, Figure 6F: rectal cancer patients, Figure 6I: CRC patients). CHGA showed significantly lower expression in CRC patients than normal controls and behaved well in the diagnostic test (AUC: 0.995). However, CHGA may not be served as a prognostic biomarker for CRC patients (*p*-values on survival test: 0.24, 0.38, and 0.13, respectively).

## 4. Discussion

Colon cancer is one of the most common types of cancers, and patients with advanced stage cancer have a poor prognosis [31]. Colonoscopy has been considered a golden test for colon cancer diagnosis [3]. However, it is invasive and expensive, and has a limited use for earlier diagnosis. As such, the development of other diagnosis methods is still needed. The detection of new biomarkers is extremely important in the diagnosis of colon cancer [5].

As mentioned in the introduction, both dry and wet experiments have their significant advantages. However, dry experiments are always doubted with their false positives, and wet experiments are limited by their laboratory environments. Hence, more and more scientists suggest combining the wet and dry experiments together, by which to make the results of studies more comprehensive and credible. In the present study, we used two ML methods (regression tree and SVM) to construct the biomarker prediction model based on the PPI network topology features and predicted CHGA as a novel diagnostic biomarker, which was further verified by IHC. A regression tree was used to find the best features of the PPI network, which were selected as the final features for the SVM prediction model. The kernel is an essential part of SVM. We tried eight different kernels in the SVM prediction model and tested their prediction accuracy using the ROC test. Finally, the “Bessel” kernel was selected with its 0.765 AUC.

Recently, many bioinformatics studies have used ML algorithms to predict new biomarkers [22,23]. Compared with previous studies, our present study used all the reported colon cancer biomarkers that were collected from our CBD database as training data to predict new biomarkers, which increased the credibility. Furthermore, prediction features were selected from a human PPI network that was optimized by jActiveModules, which increased the robustness. We used network topology features of the PPI network as prediction features. Compared with biological features, topology features can decrease the negative influences that are caused by sample heterogeneity and size for the predicted model [32]. There are two predicted biomarkers (CHGA and SCD2) that performed best in the diagnosis test. Interestingly, unlike the other ten predicted biomarkers connecting with each other, CHGA and SCD2 occupy independent positions on the PPI network. Furthermore, CHGA and SCD2 are both hubs on this PPI network, and they are close to the core networks of identified biomarkers. Many studies suggest that biological networks share similar features with the human social network. CHGA and SCD2 are just like heroes in the social network: they are alone but influence many other points in their small networks. As such, we predict that some biomarkers may share a similar network position in biological networks as heroes in social networks, and we call them “hero biomarkers”.

We have identified the diagnosis value of CHGA as a biomarker in colon cancer using meta-analysis based on gene expression data from RNA-seq and microarray, and most of these sequencing data were from colon cancer tissues [17]. In order to further confirm our results from big data analyses, in the present study, we performed an IHC on our CRC TMA samples to verify the diagnosis value for CHGA in colon cancers, which proved that CHGA could be a promising biomarker for CRC diagnosis. Our results indicate that the combination of ML and IHC for the protein analyses can provide a more acute prediction of biomarkers for CRC patients. In the future, we will examine the diagnosis value of CHGA in blood samples with liquid biopsy. If CHGA performed well in sequencing data from blood samples, then it could be used for large-scale primary screening in risk individuals and patients.

Biological functional analysis has been conducted to verify the prediction results. We found some overlapping enriched pathways for the predicted and reported biomarkers, which supported our results. Meanwhile, the results of biological function analysis inspire researchers to detect new biomarkers in these pathways.

Multiple biomarkers combined by several single biomarkers have been suggested to improve the diagnosis effect in many previous studies [7,18]. In the present study, we combined the 12 predicted biomarkers as multiple biomarkers and found that they showed significantly high diagnosis accuracy. Hence, we recommend that multiple biomarkers could be used further in the clinical trial.

## 5. Conclusions

We used ML to predict new biomarkers for colon cancer diagnosis based on the PPI network and found twelve candidate biomarkers, of which CHGA showed good diagnostic performance in both gene expression data and IHC. We combined these predicted biomarkers as multiple biomarkers and showed better performance than when used alone. Further, these predicted biomarkers share some pathways with reported biomarkers, and these pathways may be pivotal pathways for further biomarker discovery for colon cancers. More importantly, CHGA may be a potential diagnostic biomarker for colon cancer patients. The combination of ML and IHC for the protein analyses can provide a more acute prediction of biomarkers in early diagnosis.

## Figures and Tables

**Table 1:** Characteristics of patients and specimens that were included in the present study.

Parameters	Biopsy (*n* = 22)	Primary Tumor (*n* = 55)	Metastatic Lymph Node (*n* = 22)	Adjacent Normal Mucosa (*n* = 46)	Distant Normal Mucosa (*n* = 53)
Sex					
Male	10	27	12	23	27
Female	12	28	10	23	26
Age					
≤70 years	14	23	8	19	23
>70 years	8	32	14	27	30
Primary tumor location					
Colon	11	44	18	37	43
Rectum	11	11	4	9	10
TNM stage					
I	4	7	0	6	7
II	10	13	0	11	14
III	8	30	20	24	27
IV	0	5	2	5	5
Differentiation					
Well	2	5	1	4	5
Moderately	16	36	17	31	32
Poorly	4	14	4	11	16

## Data Availability

All data included in this study could be found in Supplementary Materials.

## Supplementary Materials

The following supporting information can be downloaded at: https://www.mdpi.com/article/10.3390/cancers14112664/s1, Table S1: Differential expression genes of colon cancer; Table S2: Reported Biomarker; Table S3: The definition of these network features; Table S4: Model topology features for the CCS-PPIN; Table S5: Dataset for Machine learning model construction; Table S6: Final test data; Table S7: GO annotation results for predicted and confirmed biomarkers.
